# The intrinsic excitability of and autophagy protein expression levels in dentate gyrus ensembles regulate fear generalization

**DOI:** 10.4103/NRR.NRR-D-24-01026

**Published:** 2025-06-19

**Authors:** Qing Lin, Tao Jin, Yang Yang, Xutian Hou, Ruyan Chen, Lan Ma, Xing Liu, Feifei Wang

**Affiliations:** 1 School of Basic Medicine Sciences, State Key Laboratory of Brain Function and Disease, MOE Frontiers Center for Brain Science, Institutes of Brain Science, Department of neurology, Pharmacology Research Center, Huashan Hospital, Fudan University, Shanghai, China; 2 Research Unit of Addition Memory, Chinese Academy of Medical Sciences (2021RU009), Shanghai, China

**Keywords:** anxiety, ATG7/5, c-Fos, dendritic spine, dentate gyrus, intrinsic excitability, memory generalization, Npas4

## Abstract

The overgeneralization of fear is associated with psychiatric disorders and cognitive decline. Recent studies have shown that engram cells in the dorsal dentate gyrus are integrated into functionally heterogeneous ensembles that are involved in contextual fear memory generalization and discrimination. However, the intracellular signals that promote fear generalization remain to be fully elucidated. In this study, we labeled and manipulated the c-Fos^+^ and Npas4^+^ ensembles in the dorsal dentate gyrus that are activated by contextual fear conditioning using a robust activity marking system. The results showed that increasing the excitability of Fos-dependent robust activity marking by overexpressing NaChBac or decreasing the excitability of Npas4-dependent robust activity marking by overexpressing Kir2.1 promoted fear memory generalization. Furthermore, CRISPR-mediated downregulation of the autophagy-related *Atg5* or *Atg7* genes in dorsal dentate gyrus neurons inhibited activation of c-Fos, but not Npas4. Knockdown of *Atg5* or *Atg7* in the Fos-dependent robust activity marking or Npas4-dependent robust activity marking ensemble led to an increase in neuronal excitability and a decrease in spine density in both ensembles. However, *Atg7* knockdown in the Fos-dependent robust activity marking ensemble promoted memory generalization, while knockdown of *Atg5* or *Atg7* in the Npas4-dependent robust activity marking ensemble increased anxiety levels. These results contribute to our understanding of how the varying plasticity of memory engrams is involved in regulating fear memory generalization and anxiety.

## Graphical abstract

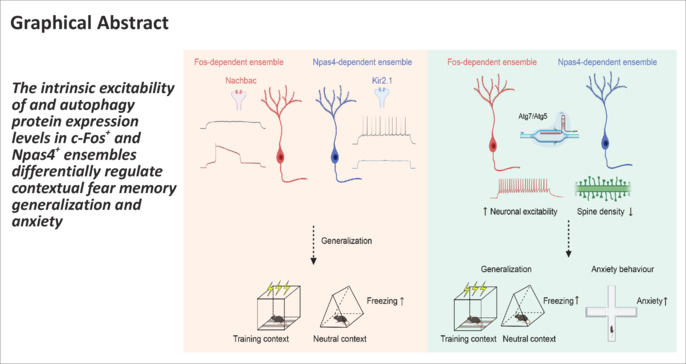

## Introduction

Memory generalization refers to a phenomenon in which an individual responds similarly to a novel stimulus that resembles a previous experience (Bergstrom, 2020). Generalization can serve an adaptive purpose aimed at survival in response to threatening stimuli (Lesuis, et al. 2021). However, overgeneralization to non-threatening cues or contexts is considered a robust correlate of certain psychiatric disorders, such as anxiety disorders, post-traumatic stress disorder (PTSD), and major depressive disorder (MDD) (Cooper et al., 2022; Hallford et al., 2022; Xuan et al., 2023; Li et al., 2024), and also characterizes cognitive decline during aging (Youm and Moscovitch, 2021; Lalani et al., 2022). Therefore, a better understanding of the neurobiological signals underlying memory generalization is fundamental to provide further insights into the treatment of anxiety disorders and aging-related cognitive impairments.

In recent years, numerous studies have supported an important role for the hippocampus in maintaining memory precision (Bian et al., 2019; Ortiz et al., 2019; Rolls, 2021; Concina et al., 2024). The dentate gyrus (DG), as the initial node of the hippocampal trisynaptic loop, performs pattern separation that orthogonalizes similar inputs from the entorhinal cortex (EC) into non-overlapping representations of information, thereby minimizing memory interference (Yassa and Stark, 2011; Hainmueller and Bartos, 2020; Wang et al., 2023). Impairments in hippocampus-dependent pattern separation have been proposed as a putative mechanism contributing to memory generalization (Nakashiba et al., 2012; Niibori et al., 2012; Becker, 2017). Memories are thought to be encoded in neuronal ensembles referred to engrams and compelling evidence suggests that distinct neuronal subpopulations within engrams can regulate the balance between memory discrimination and generalization, as indicated by studies using reporters driven by two important immediate early genes (IEGs), c-Fos and Npas4, in the dDG (Sun et al., 2020; Ren et al., 2022). c-Fos is the most well-known IEG, and it responds to a wide range of stimuli, including synaptic activity, growth factor signaling, neurotrophins, and neuromodulator signaling via cAMP (Joo et al., 2016). It is also involved in the long-term potentiation of excitatory synapses (Fleischmann et al., 2003). Unlike c-Fos, Npas4 is specifically tuned to neuronal activity (Lin et al., 2008; Bloodgood et al., 2013) and plays a key role in the regulation of inhibitory synaptic transmission (Fleischmann et al., 2003; Bloodgood et al., 2013). Current research on memory generalization predominantly focuses on specific brain regions, such as the dDG, with comparatively fewer studies examining specific neuronal ensembles. Distinct neuronal ensembles play different roles in memory. For example, Tanaka et al. (2018) demonstrated that distinct neuronal ensembles in the hippocampus separately encode spatial and contextual information. Although a study by Sun et al. (2020) investigated the mechanisms underlying synaptic plasticity in the c-Fos and Npas4 transcriptional dependent (F-RAM and N-RAM) ensembles, the contribution of other important mechanisms, such as intrinsic excitability and protein degradation, has not been investigated.

Neuronal excitability contributes to the formation and maintenance of memory engrams (Titley et al., 2017). Previous studies have shown that neurons with higher excitability are more likely to be allocated to memory engrams (Jung et al., 2023; Delamare et al., 2024; Mocle et al., 2024). Learning is known to increase neuronal excitability, which is partially mediated by the learning-induced expression of the transcription factor CREB (Oh and Disterhoft, 2015; Rashid et al., 2016; Park and Kaang, 2024). Memory recall also induces a transient increase in engram excitability, which is associated with enhanced context recognition (Pignatelli et al., 2019).

Memory formation and consolidation require protein synthesis and degradation (Kaang et al., 2009; Cuccovia et al., 2022). Autophagy is an important protein degradation pathway whereby damaged organelles and dysfunctional proteins are engulfed by autophagosomes and then delivered to lysosomes for degradation (Aman et al., 2021; Pabon et al., 2025). This process is orchestrated by multiple autophagy-related proteins (Atgs), such as Beclin1, ATG5, and ATG7 (Li et al., 2020). Increasing evidence shows that autophagy is essential for regulating important physiological functions in the mammalian brain, such as neurodevelopment, neurotransmission, and synaptic plasticity (Hernandez, et al., 2012; Compans et al., 2021; Zhang et al., 2021a; Bai et al., 2024; Lin et al., 2025). In addition, neuronal autophagy has also been implicated in memory formation, retrieval, and erasure (Shehata et al., 2018; Glatigny et al., 2019; Chen et al., 2024). Deficiencies in autophagy are observed during aging and are associated with neurodegenerative disorders such as Alzheimer’s disease (AD) and Parkinson’s disease (PD) (Lizama and Chu, 2021; Zhang et al., 2021b).

In the present study, we used a doxycycline (Dox)-dependent robust activity marking (RAM) system with a contextual fear conditioning (CFC) model, as well as electrophysiology and immunostaining, to investigate whether F-RAM and N-RAM ensemble excitability and neuronal autophagy play different roles in fear generalization.

## Methods

### Animals

C57BL/6J wild-type male mice (8–10 weeks old, 20–25 g) were obtained from the Shanghai Laboratory Animal Center (CAS, Shanghai, China, license No. SCXK (Hu) 2024-0003). Male rodents have more stable hormone levels compared with female rodents, whose estrous cycle may introduce fluctuations and affect experimental outcomes (Pestana and Graham, 2024). All mice were housed five per cage and kept on a reverse light–dark cycle (lights off at 8:00 a.m., lights on at 8:00 p.m.) at 22–26°C with ad libitum access to food and water. All mice were handled, and all experimental procedures were conducted, in accordance with the National Institutes of Health Guide for the Care and Use of Laboratory Animals (8^th^ ed., National Research Council, 2011). These procedures were approved by the Animal Care and Use Committee of the School of Basic Medical Sciences at Fudan University (approval No. 20211020-001, approval date October 20, 2021).

### Viral constructs

To generate the pAAV-F-RAM-d2tTA-TRE-Cre and pAAV-N-RAM-d2tTA-TRE-Cre plasmids, we inserted mKate2 into AAV2/8-F-RAM-d2tTA-TRE-mKate2 (Addgene: 140274) or AAV2/8-N-RAM-d2tTA-TRE-mKate2 (Addgene: 140275) using the Cre sequence obtained by PCR from pAAV-Cre-EGFP (Addgene: 68544). AAV-EF1a-DIO-EYFP and AAV-EF1a-DIO-Kir2.1-EYFP were purchased from Obio Technology Co., Ltd (Shanghai, China). AAV-EF1α-DIO-EGFP, AAV-EF1α-DIO-NaChBac-P2A-EGFP, AAV-hSyn-CRE-EGFP, AAV-EF1α-DIO-Ypet-2A-mGFP, AAV-CMV-FLEX-hSacas9-HA-Scramble-sgRNA (Scramble), AAV-CMV-FLEX-hSacas9-HA-Atg7-sgRNA (5′-GTC TGC CCA CCC GCT TGA CGT-3′), and AAV-CMV-FLEX-hSacas9-HA-Atg5-sgRNA (5′-GTC TGC CCA CCC GCT TGA CGT-3′) were purchased from BrainVTA Co., Ltd (Wuhan, Hubei, China).

### Stereotactic viral injection

Mice were anesthetized with isoflurane via inhalation using an anesthesia instrument (3.5% induction, 1.5%–2% maintenance, RWD, China) and placed in a stereotactic instrument (Stoelting, Kiel, WI, USA). AAV virus microinjections were performed with 33-gauge needles connected to a 10-μL Hamilton syringe (Hamilton, Bonaduz, Switzerland). Virus was injected bilaterally (200–300 nL per hemisphere) into the dDG at the following coordinates (Paxinos and Franklin, 2019): anterioposterior (AP) = –1.9 mm from bregma; lateral (ML) = ±1.2 mm; depth (DV) = 2.1 mm. The virus was infused at a rate of 100 nL per minute, and the needle was kept in place for 5 minutes after injection to ensure even distribution of the virus before being slowly withdrawn. Mice were allowed to recover for 3 weeks before the experiments. For experiments with Dox-dependent ensemble labeling, the animals were kept on a Dox diet (40 mg/kg; Sigma, St. Louis, MO, USA, D3447).

### Contextual fear conditioning

Mice were handled in a holding room for 3 days. For experiments with Dox-dependent ensemble labeling, the mice were taken off the Dox diet 48 hours before contextual fear conditioning. On the training day, mice were transported to the holding room and allowed to acclimate for at least 1 hour before starting the experiment. Mice were conditioned in Context A, which was a 30 cm length × 25 cm width × 33 cm height chamber with stainless-steel floors connected to a shock generator. Mice were allowed to explore the chamber freely for 3 minutes and received three shocks (0.75 mA, 2 seconds duration) at 180, 240, and 300 seconds, respectively. The mice were removed from the chamber 60 seconds after termination of the third shock, and the chamber was cleaned with 75% ethanol. After the conditioning training, the mice were returned to the Dox diet. Three days after training, the mice were placed back in training Context A and a neutral Context C (a triangular chamber with a white plastic floors and grey cover) for memory recall for 3 minutes. Freezing time was analyzed over the 3-minute exposure period. Discrimination index (DI) = (freezing time in Context A – freezing time in Context C)/(freezing time in Context A + freezing time in Context C).

### Open field test

Mice were placed in the center of an open field chamber (43.2 cm length × 43.2 cm width × 30.5 cm height; Med-Associates, St. Albans, VT, USA) and allowed to freely explore for 30 minutes. The mice were monitored by an overhead camera. The videos were analyzed using the VisuTrack system (Xinruan, Shanghai, China), and overall motor activity was quantified by measuring the total distance traveled.

### Elevated plus maze test

The elevated plus maze consisted of four arms (34.5 cm length × 6.3 cm width × 19.5 cm height) extending from a central square and was placed 75 cm above the floor. Two closed arms were delimited by 20-cm-high vertical walls, whereas the two open arms had 0.8-cm-high edges. Mice were placed in the center, and their behavior was recorded for 6 minutes using a camera located above the maze. The videos were analyzed using the VisuTrack system (Xinruan), and the cumulative time spent in the open arms was calculated.

### Immunofluorescence staining

For experiments examining IEG expression, animals were sacrificed 1.5 hours after contextual fear conditioning. The mice were perfused with 0.9% saline followed by 4% paraformaldehyde (PFA; dissolved in 1× PBS). The brains were isolated and post-fixed in 4% FFA at 4°C for 4 hours and then dehydrated in 30% sucrose for 3 days. Next, 30-μm cryosections were obtained using a cryostat microtome (Leica CM3050S, Nussloch, Germany) and washed in PBS for 10 minutes, three times. For immunofluorescence staining, the slices were incubated with primary antibody in blocking buffer (PBS containing 0.3% Triton X-100 and 10% normal donkey serum) overnight at 4°C. The next day, the brain slices were washed in PBS for 10 minutes, three times and then incubated with secondary antibody for 1.5 hours at room temperature. Finally, the slices were washed in PBS for 10 minutes, three times and mounted on slides using DAPI (Beyotime, Shanghai, China) for 5 minutes. The antibodies used were as follows: rat anti-c-Fos (1:500, Synaptic Systems GmbH, Göttingen, Germany, Cat# 226017, RRID: AB_2864765), rabbit anti-Npas4 (1:500, Activity Signaling, Beijing, China, Cat# AS-Ab18A), rabbit anti-HA (1:500, Sigma, Cat# H6908, RRID: AB_260070), goat anti-rabbit 488 (1:500, Jackson ImmunoResearch Laboratories, West Grove, PA, USA, Cat#111-545-144, RRID: AB_2338052), goat anti-rabbit Cy3 (1:500, Jackson ImmunoResearch Laboratories, Cat# 111-165-144, RRID: AB_2338006), and donkey anti-rat Alexa Fluor^TM^ 647 (1:500, Jackson ImmunoResearch Laboratories, Cat# 712-605-150, RRID: AB_2340693).

### Single-molecule RNA fluorescence *in situ* hybridization

Frozen brains were sectioned into 10-μm-thick slices and mounted on Colorfrost Plus slides (Thermo Fisher Scientific, Waltham, MA, USA). The slices were treated with hydrogen peroxide for 10 minutes at room temperature, and then subjected to target retrieval for 5 minutes at 95°C and proteolysis for 30 minutes at 40°C using RNAscope® 2.5 Universal Pretreatment Reagents (Advanced Cell Diagnostics (ACD), Newark, CA, USA, Cat# 322380). Finally, they were hybridized with probes at 40°C for 2 hours. The following probes were used for the examined genes: *Atg7*-C2 (ACD, Cat# 561261), *Atg5*-C1 (ACD, Cat# 409722), *Egfp*-C1 (ACD, Cat# 400281), and *Egfp*-C2 (ACD, Cat# 400281). After hybridization, we amplified the signals using an RNAscope Multiplex Fluorescent Reagent Kit v2 (ACD: Cat# 323110).

### Images analysis and quantification

Fluorescent images were acquired using a Nikon-A1 confocal laser scanning microscope (Nikon, Tokyo, Japan) with 20× or 60× oil-immersion objectives. To characterize c-Fos and Npas4 expression, the number of c-Fos^+^ or Npas4^+^ cells in each brain region from three to five slices per mouse (*n* = 4–6 mice per group) was manually counted using ImageJ (version 1.6.0, NIH, Bethesda, MD, USA). The boundaries of each brain region were outlined to specify regions of interest (ROIs), and the area was quantified by applying scale calibration. To determine the density of c-Fos^+^ or Npas4^+^ cells, we divided the number of positive cells by the area of the ROI. smFISH images were captured using a 20× objective lens with 3× optical zoom. The integrated intensity of *Atg7* and *Atg5* transcripts in the = ROI was analyzed using ImageJ.

To quantify dendritic spines, coronal sections were cut at 50-μm thickness, and confocal z-stack images were acquired using a 60× oil objective lens with 3× optical zoom. Confocal z-stack (1024 resolution) images with a 0.5-μm step size were taken centered on the dendritic segment. Reconstruction and analysis of the dendritic spines were carried using Imaris software (Bitplane, St. Paul, MN, USA). Apical dendritic segments of dDG engrams were chosen. Fifty to eighty micrometer segments beginning > 50 μm and ending < 150 μm distal to the soma and after the first branch point were quantified. Spine types were classified in accordance with the following criteria: stubby (length < 1 μm); mushroom (length < 3 μm and maximum width [head] ≥ 2 × mean width [neck]); and thin (length < 3 μm, mean width [head] ≥ mean width [neck]). To determine the spine density, 30 to 40 neurons from four mice in each group were measured, and the density of each spine type was expressed as the number of spines per μm of dendrite.

### *Ex vivo* electrophysiology recording

Coronal sections (300 μm) of the dDG were prepared. Briefly, the brains were sectioned on a vibratome (Thermo Fisher Scientific) in carbogenated (95% O_2_, 5% CO_2_) ice-cold cutting solution containing: 93 mM NMDG, 2.5 mM KCl, 1.25 mM NaH_2_PO_4_, 30 mM NaHCO_3_, 20 mM HEPES, 25 mM glucose, 2 mM thiourea, 5 mM Na-ascorbate, 3 mM Na-pyruvate, 0.5 mM CaCl_2_·4H_2_O, and 10 mM MgSO_4_·7H_2_O; 300–310 mOsm, with the pH adjusted to 7.3 with HCl. After initial recovery, the slices were transferred to carbogenated HEPES containing ACSF (92 mM NaCl, 2.5 mM KCl, 1.25 mM NaH_2_PO_4_, 30 mM NaHCO_3_, 20 mM HEPES, 25 mM glucose, 2 mM thiourea, 5 mM Na-ascorbate, 3 mM Na-pyruvate, 2 mM CaCl_2_·4H_2_O, and 2 mM MgSO_4_·7H_2_O; 300–310 mOsm, pH 7.4) and incubated for 60 minutes before recording. Whole-cell patch clamp recordings of the cells were performed in carbogenated recording ACSF (119 mM NaCl, 2.5 mM KCl, 1.25 mM NaH_2_PO_4_, 24 mM NaHCO_3_, 12.5 mM glucose, 2 mM CaCl_2_·4H_2_O, and mM 2 MgSO_4_·7H2O (300–310 mOsm, pH 7.3–7.4) with an EPC-10 amplifier and Pulse v8.78 software (HEKA Elektronik, Lambrecht/Pfalz, Germany). The pipette resistance was in the range of 5 to 7 MΩ. A current clamp was used, and membrane potentials were measured in response to intracellular injection of step currents (1000-ms duration, magnitudes ranging from -150 to 250 pA in 10-pA steps). The intracellular solution used was: 135 mM potassium-gluconate, 4 mM KCl, 2 mM NaCl, 10 mM HEPES, 4 mM EGTA, 4 mM Mg-ATP, 0.3 mM Na3-GTP, 10 mM sodium phosphocreatine (280–290 mOsm, pH 7.3). The signals were acquired at 5 kHz and filtered at 2 kHz. The series resistance was < 30 MΩ. Data were analyzed with Mini Analysis Program (Synaptosoft, Fort Lee, NJ, USA) or pCLAMP10.7 (Molecular Devices, San Jose, CA, USA) by an experimenter who was blinded to the experimental conditions.

### Statistical analysis

No statistical methods were used to predetermine sample sizes; however, our sample sizes are similar to those reported in previous publications (Sun et al., 2020; Chen et al., 2024). All experimental data were analyzed using SPSS 26 (IBM, Armonk, NY, USA) and plotted using GraphPad Prism 8.0 (GraphPad Software, San Diego, CA, USA, www.graphpad.com). The normality of the data was evaluated by Shapiro–Wilk test, and homoscedasticity was assessed by *F* test. Means of two groups were compared using the Student’s *t-*test (unpaired, two-tailed), Mann–Whitney *U* test when the data did not fit a normal distribution, or Welch’s *t*-test when the data did not satisfy the standard of homogeneity of variance. Means among more than two groups were compared using one-way analysis of variance (ANOVA) followed by Bonferroni’s *post hoc* analysis, Welch’s ANOVA test when the data did not satisfy the standard of homogeneity of variance, or Kruskal–Wallis *H* test when the data did not fit a normal distribution. Two-way ANOVA was used to compare group means when there were two or more variables, followed by Bonferroni’s *post hoc* analysis. CFC behavioral results were analyzed by two-way mixed ANOVA followed by Bonferroni’s post hoc analysis. Statistical significance was defined as *P* < 0.05. All data are presented as mean ± SEM. All statistical test results are presented in **Additional Table 1**.

## Results

### Increased excitability of the Fos-dependent robust activity marking ensemble or decreased excitability of the Npas4-dependent robust activity marking ensemble promotes fear memory generalization

The expression of many IEGs in regions such as the hippocampus is correlated with learning (Fleischmann et al., 2003). To investigate fear-induced expression of c-Fos and Npas4 in the dDG, mice were trained in a CFC paradigm; 1.5 hours later they were sacrificed, and the number of c-Fos^+^ and Npas4^+^ cells in the dDG was determined by immunostaining (**Additional Figure 1A**). The numbers of c-Fos^+^ and Npas4^+^ cells were significantly increased in the dDG after CFC compared with the naïve group that remained in their normal cages (**Additional Figure 1B** and **C**). c-Fos and Npas4 exhibited substantial co-localization in the dDG of mice that underwent CFC (**Additional** **Figure 1D** and **E**, 49.29% ± 3.28% of c-Fos^+^; 53.75% ± 5.05% of Npas4^+^). Since the ensembles defined by IEG mRNA, protein, and functional output can be very different, we next used a doxycycline (Dox)-dependent robust activity marking (RAM) system to detect c-Fos (F-RAM) and Npas4 (N-RAM) transcriptional outputs.

**Figure 1:**
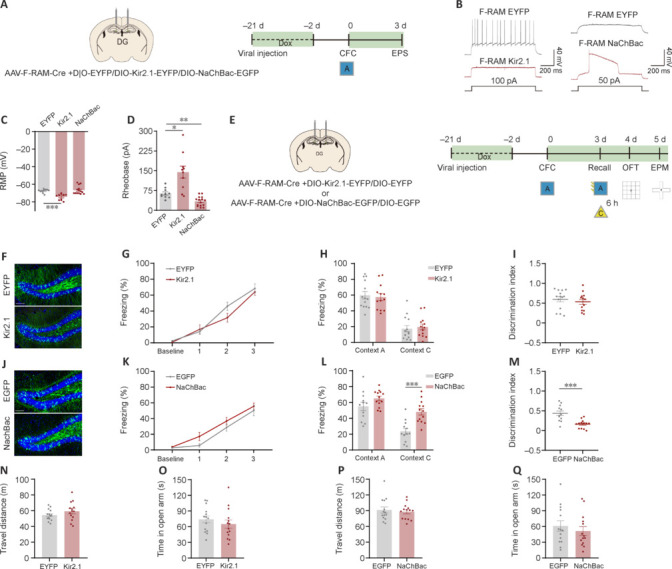
Increasing the excitability of the F-RAM promotes contextual fear memory generalization. (A) Schematic of the expression of Kir2.1 or NaChBac in the dorsal dentate gyrus F-RAM ensemble and the experimental scheme for *ex vivo* electrophysiology recording. (B) Representative action potential traces at the indicated current steps in the F-RAM ensemble. (C, D) Intrinsic electrophysiological properties: resting membrane potential and rheobase of the F-RAM ensemble after overexpressing Kir2.1 and NaChBac. EYFP: *n* = 10, Kir2.1: *n* = 10, NaChBac: *n* = 14 neurons. Resting membrane potential: Kruskal‒Wallis *H* test with the Bonferroni *post hoc* test; Rheobase: Welch’s ANOVA test with Bonferroni *post hoc* test. (E) Schematics of the expression of Kir2.1 or NaChBac in the dorsal dentate gyrus F-RAM ensemble and the experimental scheme for contextual fear memory. (F) Representative images showing the expression of EYFP and Kir2.1-EYFP in the F-RAM ensemble. Blue: DAPI; green: EGFP. Scale bar: 100 μm. EYFP: *n* = 14, Kir2.1: *n* = 13. (G–I) Freezing levels in the EYFP and Kir2.1-EYFP groups during training day (G), Context A and Context C (H) and discrimination index (I) during retrieval. Training: two-way mixed ANOVA; Context: two-way mixed ANOVA; Discrimination index: Mann‒Whitney *U* test. (J) Representative images showing the expression of EGFP and NaChBac-EGFP in the F-RAM ensemble. Blue: DAPI; green: EGFP. Scale bar: 100 μm. EGFP: *n* = 12, NaChBac: *n* = 13. (K‒M) Freezing levels in the EGFP and NaChBac-EGFP groups during training (K), in Context A and Context C (L) and the discrimination index (M) during retrieval. Training: Two-way mixed ANOVA; context: two-way mixed ANOVA with Bonferroni *post hoc* test; discrimination index: Welch’s *t* test. (N, O) Open field and elevated plus maze tests for the EYFP and Kir2.1 groups. Student’s *t*-test. (P, Q) Open field and elevated plus maze tests for the EGFP and NaChBac groups. Student’s *t*-test. The data are presented as the mean ± SEM. *P < 0.05, **P < 0.01, ***P < 0.001. ANOVA: Analysis of variance; CFC: contextual fear conditioning; DAPI: 4′,6-diamidino-2-phenylindole; EPM: elevated plus maze; EYFP: enhanced yellow fluorescent protein; F-RAM: Fos-dependent ensemble; Kir2.1: inward rectifier potassium channel 2; NaChBac: voltage-gated sodium channel; OFT: open filed test.

F-RAM and N-RAM reporter systems can be used to identify distinct subpopulations within memory engrams (Sun et al., 2020) and are likely to mediate structural and functional changes induced by Fos and Npas4, respectively.

**Figure 2:**
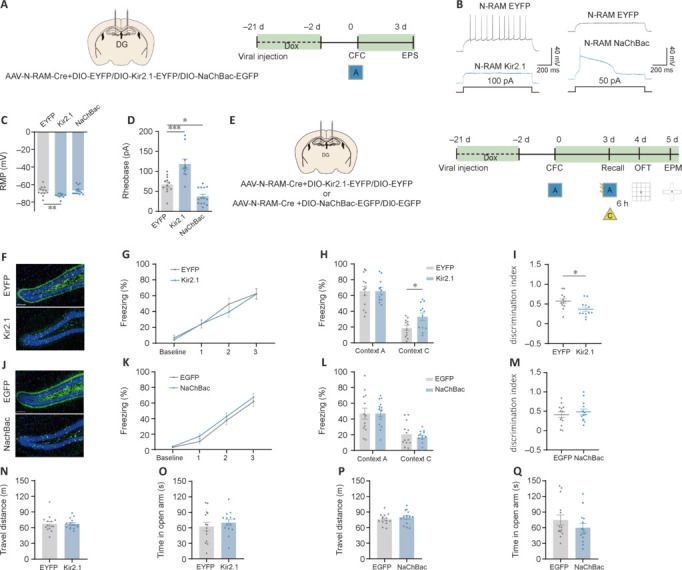
Decreasing the excitability of the N-RAM promotes contextual fear memory generalization. (A) Schematic of the expression of Kir2.1 or NaChBac in the dorsal dentate gyrus N-RAM ensemble and the experimental scheme for *ex vivo* electrophysiology recording. (B) Representative action potential traces at the indicated current steps in the N-RAM ensemble. (C, D) Intrinsic electrophysiological properties: resting membrane potential and rheobase of the N-RAM ensemble after overexpressing Kir2.1 and NaChBac. EYFP: *n* = 12, Kir2.1: *n* = 10, NaChBac: *n* = 14 neurons. Resting membrane potential: Kruskal‒Wallis *H* test with the Bonferroni *post hoc* test; Rheobase: One-way ANOVA test with the Bonferroni *post hoc* test. (E) Schematics of the expression of Kir2.1 or NaChBac in the dorsal dentate gyrus N-RAM ensemble and the experimental scheme for contextual fear memory. (F) Representative images showing the expression of EYFP and Kir2.1-EYFP in the N-RAM ensemble. Blue: DAPI; green: EGFP. Scale bar: 100 μm. EYFP: *n* = 12, Kir2.1: *n* = 12. (G–I) Levels of freezing behavior in the EYFP and Kir2.1-EYFP groups on the training day (G), Context A and Context C (H) and discrimination index (I) during retrieval. Training: two-way mixed ANOVA; context: two-way mixed ANOVA with Bonferroni *post hoc* test; discrimination index: Student’s *t-*test. (J) Representative images showing the expression of EGFP and NaChBac-EGFP in the N-RAM ensemble. Blue: DAPI; green: EGFP. Scale bar: 100 μm. EGFP: *n* = 13, NaChBac: *n* = 13. (K‒M) Freezing levels in the EGFP and NaChBac-EGFP groups during training day (K), in Context A and Context C (L) and the discrimination index (M) during retrieval. Training: two-way mixed ANOVA; Context: two-way mixed ANOVA; Discrimination index: Student’s *t*-test. (N, O) Open field tests and elevated plus maze tests for the EYFP and Kir2.1 groups. Student’s *t*-test. (P, Q) Open field tests and elevated plus maze tests for the EGFP and NaChBac groups. Student’s *t*-test and the Mann‒Whitney *U* test were used. The data are presented as the mean ± SEM. *P < 0.05, **P < 0.01, ***P < 0.001. ANOVA: Analysis of variance; CFC: contextual fear conditioning; DAPI: 4′,6-diamidino-2-phenylindole; EGFP: enhanced green fluorescent protein; EPM: elevated plus maze; EYFP: enhanced yellow fluorescent protein; Kir2.1: inward rectifier potassium channel 2; NaChBac: voltage-gated sodium channel; N-RAM: Npas4-dependent ensemble. OFT: Open filed test.

We used the inward-rectifier potassium channel Kir2.1 and the bacterial voltage sodium channel NaChBac, to decrease and increase neuronal excitability, respectively (Pignatelli et al., 2019; Hingorani et al., 2024). AAV-F/N-RAM-Cre, together with virus encoding Cre-dependent Kir2.1 or NaChBac, was injected into the mouse dDG. The mice were subjected to CFC in Context A while off Dox, and electrophysiology recordings were taken 3 days later (**Figures 1A**, **1B**, **2A**, and **2B**). Over expressing Kir2.1 channels in the F-RAM or N-RAM ensembles induced a significant decrease in the resting membrane potential (RMP) and increase in the rheobase, while overexpression of NaChBac in both ensembles induced a significant decrease in the rheobase (**Figures 1C**, **1D**, **2C**, and **2D**).

Mice overexpressing Kir2.1 or NaChBac channels in the F-RAM or N-RAM ensembles were exposed to Context A or Context C 3 days after conditioning (**Figures 1E** and **2E**). None of the manipulations affected the learning curve during CFC (**Figures 1G**, **1K**, **2G**, and **2K**). In addition to measuring freezing time in the two contexts during memory recall, we calculated the discrimination index for Context A *versus* Context C to reflect the extent of memory generalization. Overexpressing Kir2.1 in the F-RAM ensemble did not affect the freezing time in Context A and Context C (**Figure 1H**) or the discrimination index during memory recall (**Figure 1I**). Overexpressing NaChBac in the F-RAM ensemble, however, increased the freezing time in Context C (**Figure 1L**) and diminished the discrimination index (**Figure 1M**). Overexpressing Kir2.1 in the N-RAM ensemble increased the freezing level in Context C but not in Context A (**Figure 2H**) and diminished the discrimination index (**Figure 2I**), while overexpressing NaChBac in the N-RAM ensemble did not exert any effects on freezing time in Context A or Context C (**Figure 2L**) or discrimination index (**Figure 2M**).

There was no significant difference in the distance traveled in the open field test (OFT) (**Figures 1N**, **1P**, **2N**, and **2P**) and the time spent in the open arms in the elevated plus maze (EPM) (**Figures 1O**, **1Q**, **2O**, and **2Q**), indicating that overexpressing Kir2.1 or NaChBac in the ensembles did not affect locomotion or anxiety in mice. Taken together, these results suggest that either increasing F-RAM ensemble activity or decreasing N-RAM ensemble activity promotes contextual fear memory generalization.

### ATG7 and ATG5 are required for the activation of c-Fos^+^, but not Npas4^+^, neurons in the dDG

**Figure 3:**
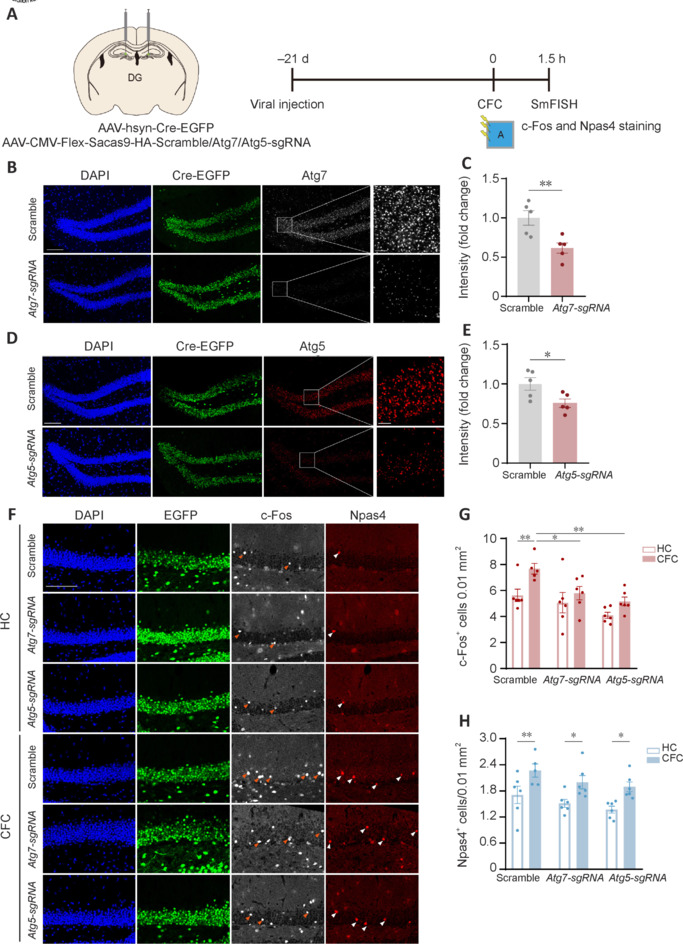
ATG7 and ATG5 are required for the activation of c-Fos but not Npas4 neurons in the dentate gyrus. (A) Schematic of the expression of *Atg7-sgRNA* and *Atg5-sgRNA* in dDG and the experimental scheme for smFISH and immunofluorescence. (B) Representative smFISH images of *Atg7* mRNA in the scramble and *Atg7-sgRNA* groups. Blue: DAPI; green: EGFP; white: Atg7. Scale bars: 100 μm, 25 μm (enlarged). (C) Quantification of *Atg7* mRNA fluorescence intensity. *n* = 5 per group. Student’s *t*-test. (D) Representative smFISH images of *Atg5* mRNA in the scramble and *Atg5-sgRNA* groups. Blue: DAPI; green: EGFP; red; *Atg5*. Scale bar: 100 μm, 25 μm (enlarged). (E) Quantification of the fluorescence intensity of the *Atg5* mRNA. *n* = 5 per group. Student’s *t*-test. (F) Representative images showing c-Fos^+^ and Npas4^+^ neurons in the dDG after contextual fear conditioning or in the home cage. Blue: DAPI; green: EGFP; white: c-Fos; red: Npas4. Scale bar: 100 μm. Arrows indicate the colabled cells. (G) Quantification of the number of c-Fos^+^ cells in the dDG after contextual fear conditioning or in the home cage. Scramble-CFC: *n* = 5; other groups: *n* = 6. Two-way ANOVA with the Bonferroni *post hoc* test. (H) Quantification of the number of Npas4^+^ in the dDG after contextual fear conditioning or under home cage Scramble-CFC: *n* = 5; other groups: *n* = 6. Two-way ANOVA with the Bonferroni *post hoc* test. The data are presented as the mean ± SEM. *P < 0.05, **P < 0.01, ***P < 0.001. ANOVA: Analysis of variance; Atg5: autophagy-related protein 5; Atg7: autophagy-related protein 7; CFC: contextual fear conditioning; DAPI: 4′,6-diamidino-2-phenylindole; dDG: dorsal dentate gyrus; EGFP: enhanced green fluorescent protein; EYFP: enhanced yellow fluorescent protein; smFISH: single-molecule RNA fluorescence in situ hybridization.

Neuronal autophagy is required for memory formation and consolidation (Shehata et al., 2018; Glatigny et al., 2019). To investigate whether autophagy-related signaling pathways in the dDG affect c-Fos and Npas4 expression during contextual fear learning, AAV vectors encoding a Cre-dependent Sacas9 and an sgRNA complementary to *Atg7* (AAV-Flex-Sacas9-HA-Atg7-sgRNA) or *Atg5* (AAV-Flex-Sacas9-HA-Atg5-sgRNA), together with AAV-hSyn-Cre (**Figure 3A**) were injected into the mouse dDG. First, *Atg7* and *Atg5* knockdown in the dDG were confirmed by single-molecule fluorescence *in situ* hybridization (smFISH). *Atg7* or *Atg5* mRNA fluorescence intensity levels in the dDG of mice expressing Sacas9 together with the corresponding sgRNA were significantly downregulated compared with control mice expressing only Sacas9 (**Figure 3B–E**). *Atg7* or *Atg5* downregulation in dDG neurons abolished the increase in c-Fos^+^ cells seen after CFC (**Figure 3F** and **G**) but had no effect on the number of Npas4^+^ cells (**Figure 3F** and **H**). These data suggest that downregulation of autophagy proteins in the dDG may affect formation of the F-RAM ensemble without affecting the N-RAM ensemble.

### ATG7 downregulation in the Fos-dependent robust activity marking ensemble promotes fear overgeneralization, whereas ATG7 or ATG5 downregulation in the Npas4-dependent robust activity marking ensemble increases anxiety

**Figure 4:**
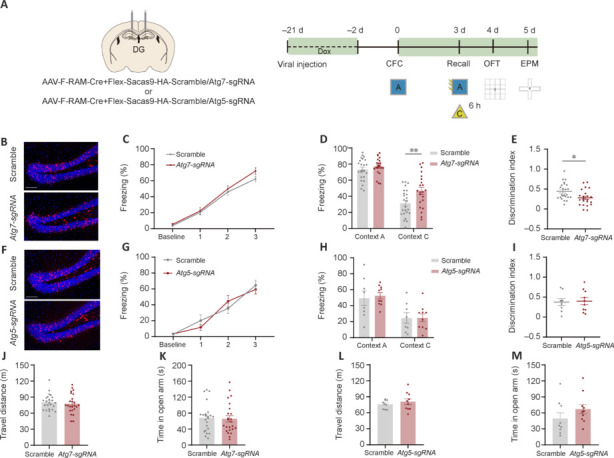
ATG7 in the F-RAM ensemble regulates contextual memory generalization. (A) Schematics of the expression of *Atg7-sgRNA* or *Atg5-sgRNA* in the dDG F-RAM ensemble and the experimental scheme for contextual fear memory. (B) Representative images showing the expression of scramble and *Atg7-sgRNA* in the F-RAM ensemble. Blue: DAPI; Red: HA. Scale bar: 100 μm. Scramble: *n* = 24, *Atg7-sgRNA*: *n* = 22. (C–E) Freezing level in the scramble and *Atg7-sgRNA* groups during training day (C), Context A and Context C (D) and discrimination index (E) during retrieval. Training: two-way mixed ANOVA; context: two-way mixed ANOVA with the Bonferroni post hoc test; discrimination index: Student’s *t* test. (F) Representative images showing the expression of scramble and *Atg5-sgRNA* in the dDG F-RAM ensemble. Blue: DAPI; Red: HA. Scale bar: 100 μm; scramble: *n* = 8; *Atg5-sgRNA*: *n* = 10. (G–I) Freezing level during conditioning (G), retrieval in Context A and Context C (H) and discrimination index (I) in the scramble and *Atg5-sgRNA* groups. Training: two-way mixed ANOVA; context: two-way mixed ANOVA; discrimination index: Student’s *t*-test. (J, K) Open field tests and elevated plus maze tests for the scramble or *Atg7-sgRNA* groups. Student’s *t*-test and the Mann‒Whitney *U* test were used. (L, M) Open field tests and elevated plus maze tests for the scramble or *Atg5-sgRNA* groups. Student’s *t*-test. The data are presented as the mean ± SEM. *P < 0.05, **P < 0.01. ANOVA: Analysis of variance; Atg5: autophagy-related protein 5; Atg7: autophagy-related protein 7; CFC: contextual fear conditioning; DAPI: 4′,6-diamidino-2-phenylindole; dDG: dorsal dentate gyrus; EPM: elevated plus maze; F-RAM: Fos-dependent ensemble; OFT: open filed test.

**Figure 5:**
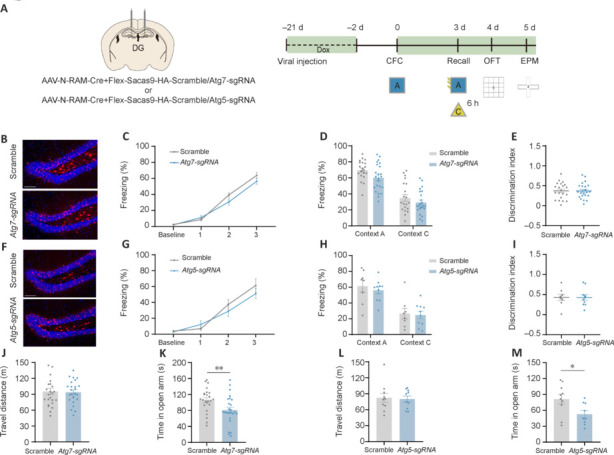
ATG7 and ATG5 in the N-RAM ensemble increased anxiety levels. (A) Schematic of the expression of *Atg7-sgRNA* or *Atg5-sgRNA* in the dorsal dentate gyrus N-RAM ensemble and the experimental scheme for contextual fear memory. (B) Representative images showing the expression of scramble and *Atg7-sgRNA* in the N-RAM ensemble. Blue: DAPI; Red: HA. Scale bar: 100 μm. Scramble: *n* = 21; *Atg7-sgRNA*: *n* = 23. (C–E) Freezing level in the scramble and *Atg7-sgRNA* groups during training day (C), Context A and Context C (D) and discrimination index (E) during retrieval. Training: two-way mixed ANOVA; context: two-way mixed ANOVA; discrimination index: Student’s *t-*test. (F) Representative images showing the expression of scramble and *Atg5-sgRNA* in the F-RAM ensemble. Blue: DAPI; Red: HA. Scale bar: 100 μm. Scramble: *n* = 8, *Atg5-sgRNA*: *n* = 10. (G–I) Freezing level in the scramble and *Atg5-sgRNA* groups during training day (G), Context A and Context C (H) and discrimination index (I) during retrieval. Training: Two-way mixed ANOVA; context: two-way mixed ANOVA; discrimination index: Student’s *t*-test. (J, K) Open field tests and elevated plus maze tests for the scramble or *Atg7-sgRNA* groups. Student’s *t*-test. (L, M) Open field tests and elevated plus maze tests for the scramble or *Atg5-sgRNA* groups. Student’s *t*-test. The data are presented as the means ± SEMs. *P < 0.05, **P < 0.01. ANOVA: Analysis of variance; Atg5: autophagy-related protein 5; Atg7: autophagy-related protein 7; CFC: contextual fear conditioning; DAPI: 4′,6-diamidino-2-phenylindole; EPM: elevated plus maze; F-RAM: Fos-dependent ensemble; N-RAM: Npas4-dependent ensemble; OFT: open filed test.

To further investigate the functional significance of autophagy pathways in F-RAM and N-RAM ensembles, AAV-Flex-Sacas9-HA-Atg7 or Atg5-sgRNA, together with AAV-F-RAM or AAV-N-RAM-Cre, were injected into the mouse dDG. After tagging the F-RAM or N-RAM ensemble with sgRNA complementary to *Atg7/5* during fear conditioning, mice were then subjected to memory recall in fear Context A or non-fear Context C, followed by the OFT and EPM tests (**Figures 4A**, **B**, **F** and **5A**, **B**, **F**). *Atg7* downregulation in F-RAM ensembles increased freezing time in Context C (**Figure 4D**) and diminished discrimination between the contexts (**Figure 4E**), whereas *Atg5* downregulation in F-RAM ensembles did not affect freezing time (**Figure 4H**) or discrimination index (**Figure 4I**). Additionally, the mice in the Atg7-sgRNA and Atg5-sgRNA groups showed similar performance in the OFT and EPM tests (**Figure 4J–M**) compared with the control group, indicating that locomotion and anxiety after fear conditioning were unaffected by knocking down *Atg7* and *Atg5* in the F-RAM ensemble.

*Atg7* or *Atg5* downregulation in the N-RAM ensemble did not affect freezing time in Context A or Context C (**Figure 5D** and **H**), the discrimination index (**Figure 5E** and **I**), or the distance traveled in the OFT test (**Figure 5J** and **L**), but did decrease the time spent exploring the open arms in the EPM test (**Figures 4C**, **G** and **5C**, **G**, **K**, **M**). These observations suggest that ATG7 in the F-RAM ensemble regulates contextual fear memory generalization, whereas both ATG7 and ATG5 in the N-RAM ensemble regulate anxiety.

### ATG7 downregulation increased Fos-dependent robust activity marking ensemble neuronal excitability, whereas ATG7 and ATG5 downregulation both increased Npas4-dependent robust activity marking ensemble neuronal excitability

**Figure 6:**
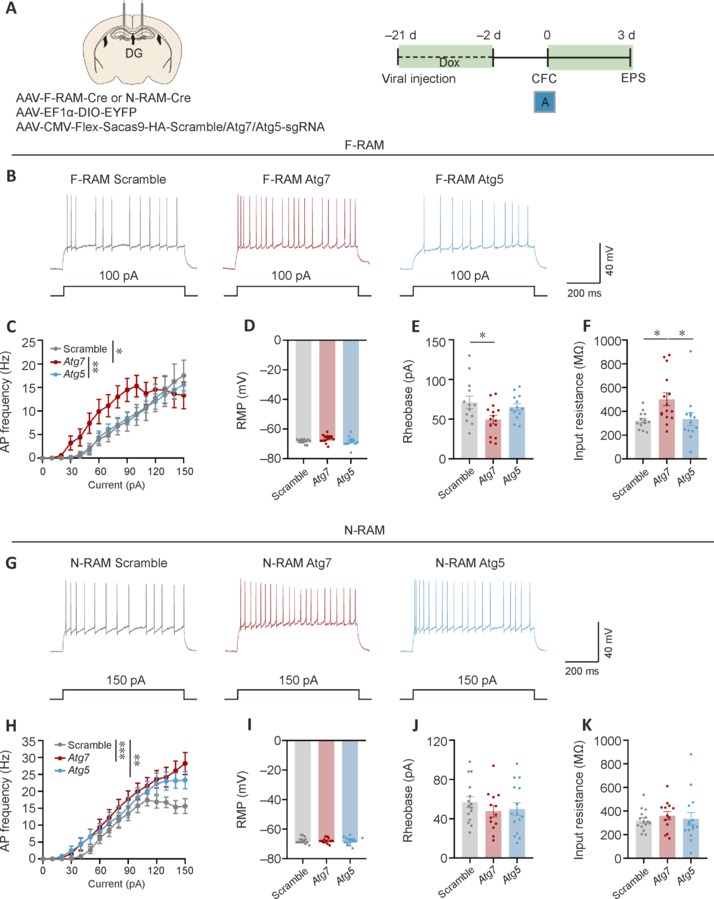
Downregulation of ATG7/5 in the F-RAM or N-RAM ensemble regulates neuronal excitability. (A) Schematic of the expression of *Atg7-sgRNA* or *Atg5-sgRNA* in the F/N-RAM ensemble and the experimental scheme for *ex vivo* electrophysiology recording. (B, C) Representative AP traces and graph of the AP frequency at the indicated current steps in the F-RAM ensemble. Scramble: *n* = 13, *Atg7-sgRNA*: *n* = 15, and *Atg5-sgRNA*: *n* = 13 neurons. Two-way mixed ANOVA with the Bonferroni *post hoc* test. (D–F) Intrinsic electrophysiological properties: RMP (D), rheobase (E), and input resistance (F) after expression of *Atg7-sgRNA* or *Atg5-sgRNA* in the F-RAM ensemble. RMP: Kruskal‒Wallis H test. Rheobase: One-way ANOVA with Bonferroni *post hoc* test; Input resistance: Kruskal‒Wallis *H* test with Bonferroni *post hoc* test. (G, H) Representative AP traces and graph of the AP frequency at the indicated current steps in the N-RAM ensemble. Scramble: *n* = 15, *Atg7-sgRNA*: *n* = 13, *Atg5-sgRNA*: *n* = 15 neurons. (I–K) Intrinsic electrophysiological properties: RMP (I), rheobase (J), and input resistance (K) after expression of *Atg7-sgRNA* or *Atg5-sgRNA* in the N-RAM ensemble. RMP: One-way ANOVA. Rheobase: One-way ANOVA; Input resistance: Kruskal‒Wallis *H* test. The data are presented as the mean ± SEM. *P < 0.05, **P < 0.01, ***P < 0.001. ANOVA: Analysis of variance; AP: action potential; Atg5: autophagy-related protein 5; Atg7: autophagy-related protein 7; CFC: contextual fear conditioning; DG: dentate gyrus; EPM: elevated plus maze; F-RAM: Fos-dependent ensemble; N-RAM: Npas4-dependent ensemble; OFT: open filed test; RMP: resting membrane potential.

To investigate the excitability of F-RAM and N-RAM ensembles after Atg7 and Atg5 knockdown, we performed *ex vivo* electrophysiology recordings 3 days after CFC (**Figure 6A**). Atg7 knockdown in F-RAM ensembles significantly increased the action potential (AP) frequency (**Figure 6B** and **C**). Knockdown of Atg7 in F-RAM ensembles did not affect RMP, whereas significant reduction in the rheobase and increase in input resistance were observed (**Figure 6D–F**). However, AP frequency, RMP, rheobase, and input resistance were not significantly altered in the F-RAM ensemble after Atg5 knockdown. A significant increase in AP frequency was observed in the N-RAM ensemble after Atg7 and Atg5 knockdown (**Figure 6G** and **H**), whereas RMP, rheobase, and input resistance in the N-RAM ensemble were not significantly changed (**Figure 6I–K**).

These results indicate that knockdown of Atg7, but not Atg5, increased membrane excitability in the F-RAM ensemble, while knockdown of Atg7 and Atg5 both increased membrane excitability in the N-RAM ensemble.

### Downregulation of ATG7 or ATG5 in Fos- and Npas4-dependent robust activity marking ensembles altered dendritic spine morphology

**Figure 7:**
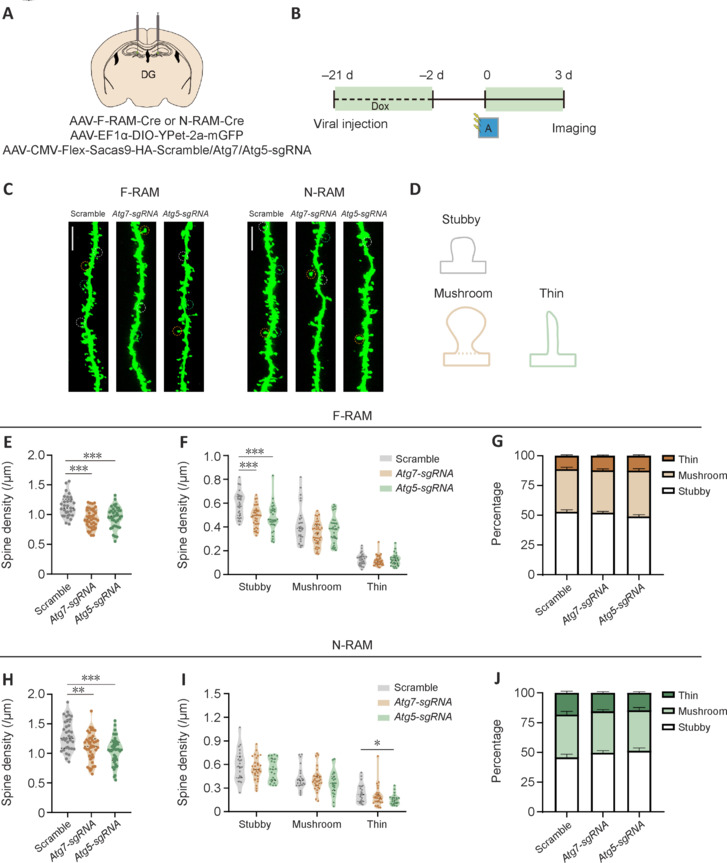
Downregulation of ATG7/5 in F-RAM or N-RAM ensembles decreased spine density and altered spine morphology (A) Schematic of the expression of Ypet-2a-mGFP and *Atg7-sgRNA* or *Atg5-sgRNA* in the dDG F-RAM or N-RAM ensemble. (B) Experimental scheme for dendritic spine imaging after contextual fear conditioning (ensemble labeling). (C) Representative images of dendritic spines in the F-RAM or N-RAM ensembles in the scramble, *Atg7-sgRNA* and *Atg5-sgRNA* groups. Green: EGFP. Scale bar: 5 μm. (D) Dendritic spine classification: stubby (gray), mushroom (orange), and thin (green) subtypes. (E) Quantification of the dendritic spine density of the F-RAM ensemble. (F) Quantification of the stubby, mushroom and thin subtype spine density of the F-RAM ensemble. (G) Quantification of the percentages of stubby, mushroom and thin spines in the F-RAM ensemble. Scramble: *n* = 28, *Atg7-sgRNA*: *n* = 40, *Atg5-sgRNA*: *n* = 32 neurons. One-way ANOVA or the Kruskal‒Wallis *H* test with Bonferroni *post hoc* test was used. (H) Quantification of the dendritic spine density of the N-RAM ensemble. (I) Quantification of the stubby, mushroom and thin spine densities of the N-RAM ensemble. (J) Quantification of the percentage of stubby, mushroom and thin spines of the N-RAM ensemble scramble: *n* = 22; *Atg7-sgRNA*: *n* = 32; and *Atg5-sgRNA*: *n*=28 neurons. One-way ANOVA or the Kruskal‒Wallis *H* test with Bonferroni *post hoc* test was used. The data are presented as the mean ± SEM. *P < 0.05, **P < 0.01, ***P < 0.001. ANOVA: Analysis of variance; Atg5: autophagy-related protein 5; Atg7: autophagy-related protein 7; dDG: dorsal dentate gyrus; F-RAM: Fos-dependent ensemble; N-RAM: Npas4-dependent ensemble.

We next asked whether downregulation of autophagy proteins in F-RAM and N-RAM ensembles affects dendritic spine density. Mice were infected with AAV-F/N-RAM-Cre and AAVs encoding Cre-dependent Ypet-2a-mGFP or Scramble/Atg7-sgRNA/Atg5-sgRNA to visualize dendritic spine structure in F-RAM and N-RAM ensembles (**Figure 7A–C**). A significant reduction in dendritic spine density in F-RAM and N-RAM ensembles in the dDG was observed in the *Atg7-sgRNA* and *Atg5-sgRNA* groups compared with the control groups expressing Scramble sgRNA (**Figure 7E** and **H**). Further classification of spine morphology into mature (stubby, mushroom) and immature (thin) spines (**Figure 7D**) showed that the proportion of each type of spine in F-RAM and N-RAM ensembles was similar in all groups (**Figure 7G** and **J**). However, the density of stubby spines in F-RAM ensembles was decreased by *Atg7-sgRNA* and *Atg5-sgRNA* (**Figure 7F**), while the density of thin spines in N-RAM ensembles was decreased by *Atg5-sgRNA* (**Figure 7I**). These results suggest that autophagy pathways are essential for fine-tuning the structural plasticity of different fear engram ensembles.

## Discussion

Our findings indicate that increasing excitability of the F-RAM ensemble and decreasing excitability of the N-RAM ensemble drive contextual fear memory generalization. Additionally, downregulation of the autophagy proteins ATG5 and ATG7 alter the spine plasticity and membrane excitability of the F-RAM and N-RAM ensembles and differentially regulate memory generalization and anxiety after fear conditioning. These findings provide novel evidence for different cell types or signaling pathways differentially modulating memory-guided behaviors.

In our study, CFC promoted activation of c-Fos and Npas4, which are critical for the formation and retrieval of contextual fear memories, in the dDG of the hippocampus (Hainmueller and Bartos, 2020), highlighting the potential importance of c-Fos and Npas4 as regulators of memory encoding and consolidation (Fleischmann et al., 2003; Ramamoorthi et al., 2011). Npas4 and c-Fos bind to the promoters of a large number of plasticity-related genes (Kim et al., 2010), indicating that they may play an important role in orchestrating downstream transcriptional programs to control cellular, synaptic, and circuit modifications of neuronal networks. Although c-Fos and Npas4 exhibited substantial co-localization in the dDG after CFC, the ensembles defined by IEG mRNA, protein, and functional output can be very different (Sun et al., 2020). For IEGs that are also transcription factors, such as c-Fos and Npas4, it is particularly important to define their engram ensembles based on their transcriptional outputs.

Excitatory and inhibitory neurotransmission play a crucial role in memory storage and processing (Jedlicka et al., 2018; Maestú et al., 2021). Disrupting the balance between excitation and inhibition in brain networks may impact the encoding, consolidating, and retrieval of fear memory, leading to altered fear responses and fear generalization (Cullen et al., 2014; Migues et al., 2016). The F-RAM ensemble receives increased excitatory inputs from the medial entorhinal cortex, whereas the N-RAM ensemble receives enhanced inhibitory inputs from local DG interneurons (Sun et al., 2020), suggesting that these two ensembles play a pivotal role in regulating the excitation–inhibition balance of neural networks and adaptive behavioral outputs. In this study, we found that increasing the intrinsic neuronal excitability of the F-RAM ensemble or decreasing the excitability of the N-RAM ensemble promoted fear memory generalization, possibly due to the excessive excitation within neural circuits. Notably, neither F-RAM nor N-RAM ensemble excitability was required for memory retrieval in the conditioned context, consistent with optogenetic inhibition of the entire dDG not impairing memory retrieval (Liu et al., 2012; Kheirbek et al., 2013). Although non-selective inhibition of all dDG neurons has no effect on memory recall, selective inhibition of a small fraction of dDG neurons (referred to as “engram cells”) impairs memory recall (Denny et al., 2014; Tonegawa et al., 2015; Josselyn and Tonegawa, 2020). The compositional heterogeneity of ensembles within the engram also raises other interesting questions, such as how the F-RAM ensemble and N-RAM ensemble interact with each other to regulate memory function and whether they interact with non-engram neurons or glia. Such interconnections between distinct ensembles remain largely unknown and will be an area of intensive investigation in the future.

Autophagy, as a pathway that delivers cellular components to lysosomes for degradation, plays an important role in synaptic plasticity and participates in memory formation, retrieval, and erasure (Shehata et al., 2018; Glatigny et al., 2019; Aman et al., 2021). In our study, we found that downregulation of *Atg7*, but not *Atg5*, in the F-RAM ensemble increased contextual fear memory generalization, whereas downregulation of *Atg7* and *Atg5* in the N-RAM ensemble did not impact fear memory generalization but did increase anxiety. Fear and anxiety are both defensive behaviors that occur in response to external stimuli. The neural circuits that contribute to fear and anxiety largely overlap (e.g., the hippocampus, amygdala, and prefrontal cortex), but our study showed that autophagy proteins in distinct neuronal ensembles differentially regulate fear memory and anxiety.

Decreased autophagy and downregulation of GABA receptor surface expression in pyramidal neurons in the prefrontal cortex lead to an imbalance in excitatory–inhibitory neurotransmission, as well as cognitive deficits (Sumitomo et al., 2018). *Atg5* knockdown in excitatory neurons results in increased GluR1 surface expression and enhances excitatory input (Overhoff et al., 2022). These reports suggest that autophagy can selectively degrade postsynaptic receptors or synaptic proteins and modulate ion channel function to regulate neuronal excitability and excitatory–inhibitory neurotransmission. The electrophysiology recordings taken in our study showed that downregulation of ATG7, but not ATG5, increased F-RAM ensemble neuronal excitability, whereas ATG7 or ATG5 downregulation both increased neuronal excitability of the N-RAM ensemble, although not to the same extent as that observed in the F-RAM ensemble. This may partially explain why downregulation of *Atg7*, but not *Atg5*, in the F-RAM ensemble increased contextual fear memory generalization, consistent with our finding that increased F-RAM ensemble excitability promotes memory generalization. However, increasing N-RAM ensemble neuronal excitability by overexpressing NaChBac caused neither fear generalization nor anxiety. We speculate that the increased anxiety induced by Atg7/5 knockdown in the N-RAM ensemble may require additional mechanisms.

A previous study showed that loss of autophagy in spiny projection neurons of the direct (dSPN) pathway in the striatum reduces dendritic spine density and inhibits excitatory input, while loss of autophagy in the indirect (iSPN) pathway increases intrinsic excitability, in part due to reduced function of the inwardly rectifying potassium channel Kir2.1 (Lieberman et al., 2020). This indicates that autophagy functions differently in different cell types, which is consistent with our findings. Indeed, in addition to regulating canonical autophagy, ATGs also participate in non-canonical autophagy, such as LC3-associated phagocytosis, and exhibit several non-autophagic functions (Shang et al., 2024). Some studies have shown that the autophagic and non-autophagic functions of ATGs are interrelated (Subramani and Malhotra, 2013; Han et al., 2014). Whether *Atg7* or *Atg5* knockdown affects their non-autophagic functions need to be assessed in the future.

There is substantial evidence supporting the critical role of dendritic spines, including their morphology and stability, in connectivity and neuronal excitability (Harms and Dunaevsky, 2007). Different morphological types of spines are associated with variations in synaptic plasticity and cognitive functions. Spines with larger heads (i.e., mushrooms and stubby types) are more stable and often form strong synapses characterized by larger presynaptic contacts, greater postsynaptic density, and increased AMPA-type glutamate receptor expression levels, while thin spines are more dynamic, transient, and plastic (Hering and Sheng, 2001). In our electrophysiological experiments, knockdown of autophagy proteins increased the excitability of both the F-RAM and N-RAM ensembles; however, knockdown of *Atg7* or *Atg5* in the F-RAM and N-RAM ensembles decreased spine density and changed spine morphology. These results may seem contradictory, but do not rule out the possibility that a reduction in dendritic spine density is a consequence of neuronal homeostatic plasticity. Homeostatic plasticity mechanisms promote compensatory changes in cellular excitability in response to chronic changes in network activity, which is crucial for maintaining brain circuit (Moulin et al., 2019, 2022). We speculate that the sustained neuronal excitation caused by knockdown of autophagy proteins leads to synaptic scaling, which in turn results in a decrease in dendritic spine density.

This study had some limitations that should be noted. Although the present study demonstrated the differential effects of manipulating ATG7 and ATG5 in F-RAM and N-RAM ensembles, we did not elucidate the specific molecular and cellular mechanisms by which these changes in autophagy protein expression levels lead to alternations in neuronal excitability and dendritic spine morphology, especially the different pathways involving Atg7 and Atg5 (including non-autophagic pathways) or potential compensatory mechanisms that could arise in response to the manipulations. In future studies, transcriptomic analysis could be used to identify downstream molecules of Atg7 and Atg5, thereby facilitating more comprehensive interpretation of our results. In addition, it is noteworthy that N-RAM ensemble excitability is necessary but not sufficient for fear memory discrimination. Npas4 is expressed in both inhibitory and excitatory neurons. It recruits inhibitory synapses on excitatory neurons, but it also strengthens excitatory synapses on inhibitory GABAergic neurons (Lin et al., 2008; Spiegel et al., 2014; Yoshihara et al., 2014). Npas4 in CA3 pyramidal neurons selectively restricts excitatory mossy fiber input from DG granule cells (Weng et al., 2018). Given the previous observation that the N-RAM ensemble is preferentially regulated by local DG interneurons (Sun et al., 2020), the potential function of modulators that regulate inhibitory synaptic connectivity need to be assessed in order to elucidate their role in N-RAM ensemble disinhibition and fear generalization. Furthermore, we did not assess behaviors at later time points, and remote memory should be assessed in future studies to determine whether the changes observed in our study are sustained over time.

In summary, our results suggest that neuronal excitability and autophagy-related pathways in different dDG ensembles are the cellular basis of fear generalization. Increased F-RAM ensemble excitability and decreased N-RAM ensemble excitability promoted contextual fear memory generalization. *Atg7* downregulation promoted memory generalization, whereas knockdown of either *Atg5* or *Atg7* in the N-RAM ensemble increased anxiety. Downregulation of *Atg7* or *Atg5* in F-RAM and N-RAM ensembles resulted in increased neuronal excitability, decreased dendritic spine density, and altered dendritic spine morphology. Overall, our study provides new insight into engram heterogeneity and its diverse functions and could be used to develop new strategies and interventions for individuals with emotional disorders like PTSD.

## Additional files:

***Additional Figure 1:*** *Contextual fear conditioning promotes the activation of c-Fos*^*+*^ *and Npas4*^*+*^ *neurons in the dDG.*

Additional Figure 1 Contextual fear conditioning promotes the activation of c-Fos^+^ and Npas4^+^ neurons in the dDG.
(A) Experimental scheme for examining the activation of c-Fos^+^ and Npas4^+^ neurons by immunostaining after CFC or HC. (B) Representative images showing c-Fos^+^ and Npas4^+^ cells in the dDG. Green: Npas4; Red: c-Fos; Blue: DAPI. Scale bar: 100 μm. (C) Quantification of the number of c-Fos^+^ and Npas4^+^ cells in the dDG after CFC. *n* = 5 per group. Student's t-test. (D) Representative images showing c-Fos^+^ and Npas4^+^ cell colocalization in the dDG. Red: Npas4; green: c-Fos; blue: DAPI. Arrows indicate colocalized cells. Scale bar: 20 μm. (E) Quantification of c-Fos^+^ and Npas4^+^ cell colocalization in the dDG. *n* = 6. The data are presented as the mean ± SEM. *P < 0.05, ***P < 0.001. CFC: Contextual fear conditioning; DAPI: 4ʹ,6-diamidino-2-phenylindole; dDG: dorsal dentate gyrus; HC: home cage.

***Additional Table 1:*** *All statistical analyses corresponding to figures.*

Additional Table 1 All statistical analyses corresponding to figures

## Data Availability

*All relevant data are within the paper and its Additional files*.
