# Supplementary material for: The intrinsic excitability of and autophagy protein expression levels in dentate gyrus ensembles regulate fear generalization
Source: Neural Regen Res. 2025 Jun 19;21(7):3073–82. doi: 10.4103/NRR.NRR-D-24-01026 (PMC13378950; doi:10.4103/NRR.NRR-D-24-01026)
Supplement: Supplementary file 1 [file NRR-21-3073_Suppl1.pdf]

Additional Table 1 All statistical analyses corresponding to figures

Figure		Experiemts	Statistical test	Statistical results	Post Hoc test
Additional Figure 1	1C	The number of c-Fos^{+} and Npas4+ neurons in the DG	Student's t-test<br>Student's t-test	c-Fos: t(8)=6.625, *P = 0.0304<br>Npas4: t(8)=5.199, ***P = 0.0008	
	1C	Resting membrane potential of the F-RAM ensemble after overexpressing Kir2.1 and NachBac	Kruskal-Wallis H test	H=17.81, ***P=0.0001	EYFP vs. Kir2.1: ***P=0.0008<br>EYFP vs. NaChBac: P>0.9999<br>Kir2.1 vs. NaChBac: ***P=0.0005
	1D	Rheobase of the F-RAM ensemble after overexpressing Kir2.1 and NachBac	Welch's ANOVA test	F(2,16.57) = 16.55, ***P<0.0001	EYFP vs. Kir2.1:*P=0.0169<br>EYFP vs. NaChBac**P=0.0029<br>Kir2.1 vs. NaChBac: ** P=0.0022
	1G	Freezing levels in context A during conditioning(%) when decreasing the excitability of the F-RAM ensemble	Two-way mixed ANOVA, Bonferroni multiple comparison test	Time: F(3,75)= 159.79,***P<0.0001<br>Treatment: F (1,25)=0.8329,P=0.3701<br>Time × Treatment:F(3,75)=2.695,P=0.0520	
	1H	Freezing levels in contexts A and C (%) when decreasing the excitability of the F-RAM ensemble	Two-way mixed ANOVA, Bonferroni multiple comparison test	Context: F(1, 25) = 175.9,***P<0.0001<br>Treatment: F(1,25)=0.0001, P=0.9920<br>Contex × Treatment: F(1, 25) = 0.5936, P=0.4483	
Figure 1	1I	Discrimination indices in contexts A and C when decreasing the excitability of the F-RAM ensemble	Mann-Whitney U test	U=75, P=0.4583	
	1K	Freezing levels in context A during conditioning (%) when increasing the excitability of the F-RAM ensemble	Two-way mixed ANOVA, Bonferroni multiple comparison test	Time: F(3,69)= 94.87,***P<0.0001<br>Treatment: F (1,23)=2.261, P=0.1462<br>Time × Treatment: F(3,69)=0.9179, P=0.4369	
	1L	Freezing levels in contexts A and C (%) when increasing the excitability of the F-RAM ensemble	Two-way mixed ANOVA, Bonferroni multiple comparison test	Context: F(1, 23) = 84.26,***P<0.0001<br>Treatment: F(1,23)=11.43, **P=0.0026<br>Context × Treatment: F(1, 23) = 7.591, *P=0.0113	A:EGFP vs. NachBac: P=0.1055<br>B:EGFP vs. NachBac: ***P=0.0002
	1M	Discrimination indices in contexts A and C when increasing the exitability of the F-RAM ensemble	Welch's t-test	t(16.393)=4.124,***P=0.0008	
	1N	Open field tests for EYFP or Kir2.1 groups (F-RAM)	Student's t-test	t(26)=1.331, P=0.1947	
	1O	Elevated plus maze tests for EYFP or Kir2.1 groups((F-RAM)	Student's t-test	t(26)=0.8861, P=0.3837	
	1P	Open field tests for EGFP or NachBac groups(F-RAM)	Student's t-test	t(26)=0.4445, P=0.6603	
	1Q	Elevated plus maze tests for EGFP or NachBac groups(F-RAM)	Student's t-test	t(26) =0.7657, P=0.4508	
	2C	Resting membrane potential of the N-RAM ensemble after overexpressing Kir2.1 and NachBac	Kruskal-Wallis H test	H=13.56, **P=0.0011	EYFP vs. Kir2.1:**P=0.0053<br>EYFP vs. NaChBac:P>0.9999<br>Kir2.1 vs. NaChBac:**P=0.0023
	2D	Rheobase of the N-RAM ensemble after overexpressing Kir2.1 and NachBac	One-way ANOVA,Bonferroni multiple comparison test	F(2,33) = 25.86, ***P<0.0001	EYFP vs. Kir2.1:***P=0.0003<br>EYFP vs. NaChBac: *P=0.0262<br>Kir2.1 vs. NaChBac:***P<0.0001
	2G	Freezing levels in context A during conditioning(%) when decreasing the excitability of the N-RAM ensemble	Two-way mixed ANOVA, Bonferroni multiple comparison test	Time: F(3,66)= 66.705,***P<0.0001<br>Treatment: F(1,22)=0.3022, P=0.5881<br>Time × Treatment: F(3,66)=0.6035, P=0.6150	
	2H	Freezing levels in contexts A and C (%) when decreasing the excitability of the N-RAM ensemble	Two-way mixed ANOVA, Bonferroni multiple comparison test	Context: F(1, 22) = 149.258,***P<0.0001<br>Treatment: F(1,22)=1.554, P=0.2257<br>Context ×Treatment: F(1,22) = 4.811, *P=0.0391	A:EYFP vs. Kir2.1 :P=0.9967<br>B:EYFP vs. Kir2.1:*P=0.0217
Figure 2	2I	Discrimination indices in contexts A and C when decreasing the excitability of the N-RAM ensemble	Student's t-test	t(22)=2.580,*P=0.0171	

	2K	Freezing levels in context A during conditioning(%) when increasing the excitability of the F-RAM ensemble	Two-way mixed ANOVA, Bonferroni multiple comparison test	Time: F(2.243,72)= 149.077,***P<0.0001<br>Treatment: F (1,24)=1.393, P=0.2495<br>Time × Treatment:F(2.243,72)=0.3247, P=0.7485	
	2L	Freezing levels in contexts A and C (%) when increasing the excitability of the F-RAM ensemble	Two-way mixed ANOVA, Bonferroni multiple comparison test	Context:F (1, 24) = 55.88,***P<0.0001<br>Treatment: F (1,24)=0.126, P=0.7255<br>Context × Treatment:F (1, 24) = 0.208, P=0.6521	
	2M	Discrimination indices in contexts A and C when increasing the excitability of the F-RAM ensemble	Student's t-test	t(24)=0.7165,P=0.4806	
	2N	Open field tests for EYFP or Kir2.1 groups(N-RAM)	Student's t-test	t(26)=0.0453, P=0.9642	
	2O	Elevated plus maze tests for EYFP or Kir2.1 groups (N-RAM)	Student's t-test	t(26)=0.7246, P = 0.4752	
	2P	Open field tests for EGFP or NachBac groups (N-RAM)	Student's t-test	t(26)=0.8145, P=0.4228	
	2Q	Elevated plus maze tests for EGFP or NachBac groups (N-RAM)	Mann-Whitney U test	U=71.5, P=0.2319	
	3C	*Atg7* mRNA fluorescence intensity	Student's t-test	t(8)=3.372, P=**0.0098	
	3E	*Atg5* mRNA fluorescence intensity	Student's t-test	t(8)=2.541, *P=0.0347	
**Figure 3**	3G	The number of c-Fos+ neurons in the DG	Two-way ANOVA, Bonferroni multiple comparison test	Context: F(1, 29) = 9.919, **P=0.0038<br>Treatment: F(2,29)=8.186 , **P=0.0015<br>Context × Treatment: F(2, 29) = 0.9170, P=0.4110	Scramble: HC vs. CFC,** P=0.0087;<br>Atg7-sgRNA: HC vs. CFC, P=0.3028;<br>Atg5-sgRNA: HC vs. CFC, P=0.1332<br><br>HC: Scramble vs. Atg7-sgRNA, P>0.9999;<br>Scramble vs. Atg5-sgRNA, P= 0.1041;<br>CFC: Scramble vs. Atg7-sgRNA,<br>*P=0.0454; Scramble vs. Atg5-sgRNA,**<br>P=0.0051
	3H	The number of Npas4+ neurons in the DG	Two-way ANOVA, Bonferroni multiple comparison test	Context: F(1, 29) = 21.71,***P<0.0001<br>Treatment: F(2,29)=3.398 , *P=0.0472<br>Context × Treatment: F(2, 29) = 0.0371, P=0.9636	Scramble: HC vs. CFC, **P=0.0092;<br>Atg7-sgRNA: HC vs. CFC, *P=0.0169;<br>Atg5-sgRNA: HC vs. CFC, *P=0.0104;<br><br>HC: Scramble vs. Atg7-sgRNA, P=0.9432;<br>Scramble vs. Atg5-sgRNA, P= 0.2602;<br>CFC: Scramble vs. Atg7-sgRNA,<br>P=0.5603; Scramble vs. Atg5-sgRNA, P=<br>0.2147
	4C	Freezing levels in context A during conditioning(%) when knockdown of Atg7 in the F-RAM ensemble	Two-way mixed ANOVA, Bonferroni multiple comparison test	Time: F(3,132)= 200.7,***P<0.0001<br>Treatment: F (1,44)=1.612, P=0.2108<br>Time × Treatment:F(3,132)=1.001, P=0.3946	
	4D	Freezing levels in contexts A and C (%) when knockdown of Atg7 in the F-RAM ensemble	Two-way mixed ANOVA, Bonferroni multiple comparison test	Context: F(1, 44) =254.5,***P<0.0001<br>Treatment: F (1,44)=5.489 , *P=0.0237<br>Context × Treatment:F (1, 44) = 6.474,* P=0.0145	A: CNT vs. Atg7-sgRNA :P=0.2941<br>B: CNT vs. Atg7-sgRNA:**P=0.0083
	4E	Discrimination indices in contexts A and C when knockdown of Atg7 in the F-RAM ensemble	Student's t test	t(44)=2.678, *P=0.0104	
**Figure4**	4G	Freezing levels in context A during conditioning(%) when knockdown of Atg5 in the F-RAM ensemble	Two-way mixed ANOVA, Bonferroni multiple comparison test	Time: F(3,48)= 65.572,***P<0.0001<br>Treatment: F (1,16)=0.046, P=0.8336<br>Time × Treatment: F(3,48)=1.416, P=0.2496	
	4H	Freezing levels in contexts A and C (%) when knockdown of Atg5 in the F-RAM ensemble	Two-way mixed ANOVA, Bonferroni multiple comparison test	Context F(1,16) =38.95,***P<0.0001<br>Treatment: F(1,16)=0.047, P=0.8305<br>Context × Treatment: F(1,44) = 0.123, P=0.7306	
	4I	Discrimination indices in contexts A and C when knockdown of Atg5 in the F-RAM ensemble	Student's t-test	t(16)=0.1840, P=0.8563	
	4J	Open field tests for Scramble orAtg7-sgRNA groups(F-RAM)	Student's t-test	t(46)=0.5075, P=0.6124	
	4K	Elevated plus maze tests for Scramble orAtg7-sgRNA groups (F-RAM)	Mann–Whitney U test	U=260, P=0.7438	
	4L	Open field tests for Scramble or Atg5-sgRNA groups (F-RAM)	Student's t-test	t(17)=0.84, P=0.4126	
	4M	Elevated plus maze tests for Scramble orAtg5-sgRNA groups (F-RAM)	Student's t-test	t(17)=1.286, P=0.2158	

	5C	Freezing levels in context A during conditioning(%) when knockdown of Atg7 in the N -RAM ensemble	Two-way mixed ANOVA, Bonferroni multiple comparison test	Time: F(2.272,126)= 225.9,***P<0.0001<br>Treatment: F (1,42)=1.905, P=0.1750<br>Time × Treatment: F(2.272,126)=2.402, P=0.0889	
	5D	Freezing levels in contexts A and C (%) when knockdown of Atg7 in the N-RAM ensemble	Two-way mixed ANOVA, Bonferroni multiple comparison test	Context: F(1, 42) =201.40,***P<0.0001<br>Treatment: F(1,42)=3.338, P=0.0748<br>Context × Treatment: F(1,42) = 0.740, P=0.3947	
	5E	Discrimination indices in contexts A and C when knockdown of Atg7 in the N-RAM ensemble	Student's t-test	t(41)=0.0683, P=0.9459	
**Figure5**	5G	Freezing levels in context A during conditioning(%) when knockdown of Atg5 in the N-RAM ensemble	Two-way mixed ANOVA, Bonferroni multiple comparison test	Time: F(3,48)= 64.894, ***P<0.0001<br>Treatment: F (1,16)=0.456, P=0.5090<br>Time × Treatment: F(3,48)=1.46, P=0.2371	
	5H	Freezing levels in contexts A and C (%) when knockdown of Atg5 in the N-RAM ensemble	Two-way mixed ANOVA, Bonferroni multiple comparison test	Context: F (1, 16) =72.065,***P<0.0001<br>Treatment: F (1,16)=0.249, P=0.6244<br>Context × Treatment: F(1,16) = 0.209, P=0.6540	
	5I	Discrimination indices in contexts A and C when knockdown of Atg5 in the N-RAM ensemble	Student's t-test	t(16)=0.025, P=0.9804	
	5J	Open field tests for Scramble orAtg7-sgRNA groups(N-RAM)	Student's t-test	t(47)=0.1732, P=0.8632	
	5K	Elevated plus maze tests for Scramble orAtg7-sgRNA groups (N-RAM)	Student's t-test	t(46)=2.968, **P=0.0047	
	5L	Open field tests for Scramble or Atg5-sgRNA groups (N-RAM)	Student's t-test	t(18)=0.2275, P=0.8226	
	5M	Elevated plus maze tests for Scramble orAtg5-sgRNA groups (N-RAM)	Student's t-test	t(17)=2.443, *P=0.0258	
	6C	AP frequency at the indicated current steps for Scramble, Atg7-sgRNA or Atg5-sgRNA groups （F-RAM ）	Two-way mixed ANOVA, Bonferroni multiple comparison test	Time: F(1.984, 75.41) = 47.09, P<0.0001<br>Treatment: F(2, 38) = 1.492,P=0.2378<br>Time × Treatment: F(30, 570) = 1.681, *P=0.014	Scramble vs. Atg7: *P=0.0119<br>Scramble vs. Atg5: P>0.9999<br>Atg7 vs. Atg5: **P=0.0015
	6D	Resting membrane potential for Scramble, Atg7-sgRNA or Atg5-sgRNA groups （F-RAM ）	Kruskal-Wallis H test	H=6.395, *P=0.0409	Scramble vs. Atg7: P=0.1227<br>Scramble vs. Atg5: P>0.9999<br>Atg7 vs. Atg5: P=0.0701
	6E	Rheobase for Scramble, Atg7-sgRNA or Atg5-sgRNA groups（F-RAM ）	One-way ANOVA,Bonferroni multiple comparison test	F (2, 38) = 3.835,*P=0.0304	Scramble vs. Atg7: *P=0.0344<br>Scramble vs. Atg5: P>0.9999<br>Atg7 vs. Atg5: P=0.1847
	6F	Input resistance for Scramble, Atg7-sgRNA or Atg5-sgRNA groups（F-RAM ）	Kruskal-Wallis H test	H=8.718,*P=0.0128	Scramble vs. Atg7: *P=0.0411<br>Scramble vs. Atg5: P>0.9999<br>Atg7 vs. Atg5: *P=0.0293
**Figure6**	6H	AP frequency at the indicated current steps for Scramble, Atg7-sgRNA or Atg5-sgRNA groups （N-RAM ）	Two-way RM ANOVA, Bonferroni multiple comparison test	Time: F(2.570, 102.8) = 120.0, P<0.0001<br>Treatment: F (2, 40) = 1.977, P=0.1518<br>Time × Treatment: F (30, 600) = 1.836, **P=0.0047	Scramble vs. Atg7: ***P=0.0001<br>Scramble vs. Atg5: **P=0.0061<br>Atg7 vs. Atg5: P=0.8341
	6I	Resting membrane potential for Scramble, Atg7-sgRNA or Atg5-sgRNA groups （N-RAM ）	One-way ANOVA,Bonferroni multiple comparison test	F(2, 40) = 0.06510, P=0.9371	
	6J	Rheobase for Scramble, Atg7-sgRNA or Atg5-sgRNA groups（N-RAM ）	One-way ANOVA,Bonferroni multiple comparison test	F(2, 40) = 0.6392, P=0.5330	
	6K	Input resistance for Scramble, Atg7-sgRNA or Atg5-sgRNA groups（N-RAM ）	Kruskal-Wallis H test	H=1.549, P=0.4610	
	7E	Total spine density when knockdown of Atg7 or Atg5 in the F -RAM ensemble	One-way ANOVA,Bonferroni multiple comparison test	F(2,107)=13.53,***P<0.0001	Scramble vs. Atg7-sgRNA:***P<0.0001<br>Scramble vs. Atg5-sgRNA:***P<0.0001<br>Atg7-sgRNA vs. Atg5-sgRNA: P>0.9999
		Stubby type density when knockdown of Atg7 or Atg5 [illegible]	One-way ANOVA,Bonferroni multiple comparison [illegible]		Scramble vs. Atg7-sgRNA:***P=0.0002

		Stubby type density when knockdown of Atg7 or Atg5 in the F -RAM ensemble	One-way ANOVA,Bonferroni multiple comparison test	F(2,97)=12.23,***P<0.0001	Scramble vs. Atg5-sgRNA:***P<0.0001<br>Atg7-sgRNA vs. Atg5-sgRNA: P>0.9999
	7F	Mushroom type density when knockdown of Atg7 or Atg5 in the F -RAM ensemble	Kruskal-Wallis H test	H=4.446, P=0.1083	
		Thin type density when knockdown of Atg7 or Atg5 in the F -RAM ensemble	Kruskal-Wallis H test	H=1.374, P=0.5031	
		The percentage of stubby when knockdown of Atg7 or Atg5 in the F -RAM ensemble	One-way ANOVA,Bonferroni multiple comparison test	F(2,97)=2.241, P=0.1111	
	7G	The percentage of mushroom when knockdown of Atg7 or Atg5 in the F -RAM ensemble	Kruskal-Wallis H test	H=2.476, P=0.2900	
**Figure 7**		The percentage of thin when knockdown of Atg7 or Atg5 in the F -RAM ensemble	One-way ANOVA,Bonferroni multiple comparison test	F(2,97)=0.9534, P=0.3890	
	7H	Total spine density when knockdown of Atg7 or Atg5 in the N-RAM ensemble	One-way ANOVA,Bonferroni multiple comparison test	F(2,80)=9.183,***P=0.0003	Scramblevs Atg7-sgRNA: **P=0.0078<br>Scramblevs Atg5-sgRNA: ***P=0.0002<br>Atg7-sgRNA vs. Atg5-sgRNA: P=0.6215
		Stubby type density when knockdown of Atg7 or Atg5 in the N-RAM ensemble	One-way ANOVA,Bonferroni multiple comparison test	F(2,73)=0.9270, P=0.4003	
	7I	Mushroom type density when knockdown of Atg7 or Atg5 in the N-RAM ensemble	Kruskal-Wallis H test	H=1.438, P=0.4872	
		Thin type density when knockdown of Atg7 or Atg5 in the N-RAM ensemble	Kruskal-Wallis H test	H=6.996, *P=0.03	Scramblevs Atg7-sgRNA:P=0.1410<br>Scramblevs Atg5-sgRNA:*P=0.0327<br>Atg7-sgRNA vs. Atg5-sgRNA: P>0.9999
		The percentage of stubby when knockdown of Atg7 or Atg5 in the N-RAM ensemble	One-way ANOVA,Bonferroni multiple comparison test	F(2,73)=0.9651, P=0.3857	
	7J	The percentage of mushroom when knockdown of Atg7 or Atg5 in the N-RAM ensemble	One-way ANOVA,Bonferroni multiple comparison test	F(2,73)=0.0406, P=0.9603	
		The percentage of thin when knockdown of Atg7 or Atg5 in the N-RAM ensemble	Kruskal-Wallis H test	H=2.885, P=0.2363	
