# Supplementary figures and images for: The intrinsic excitability of and autophagy protein expression levels in dentate gyrus ensembles regulate fear generalization
Source: Neural Regen Res. 2025 Jun 19;21(7):3073–82. doi: 10.4103/NRR.NRR-D-24-01026 (PMC13378950; doi:10.4103/NRR.NRR-D-24-01026)

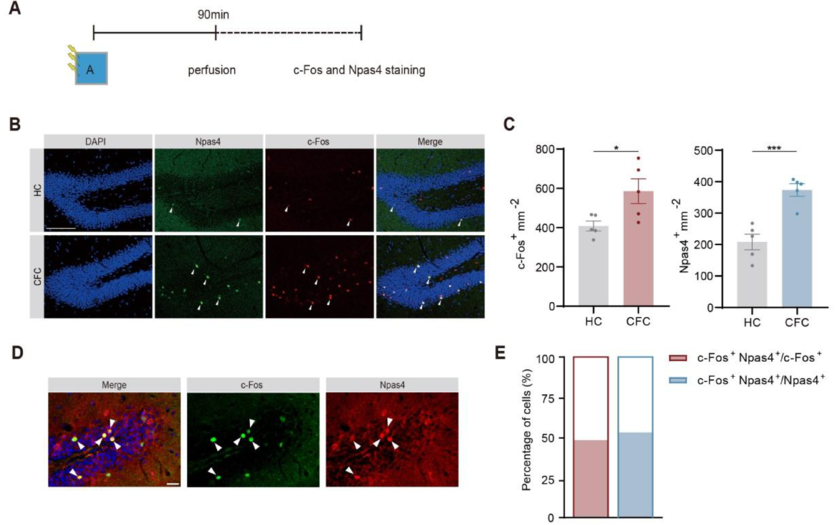

Supplement: Supplementary file 2 [file NRR-21-3073_Suppl1.tif]
